# Investigation of Natural Refractive Changes in Young Hyperopic Children Using Atropine Cycloplegic Refraction

**DOI:** 10.7759/cureus.96440

**Published:** 2025-11-09

**Authors:** Toru Kawanobe, Toshiaki Goseki, Shinya Takahashi, Shingo Noda, Yasuhito Ikeda, Eri Ishikawa, Yuichiro Tanaka, Tadahiko Kozawa

**Affiliations:** 1 Ophthalmology, Kozawa Eye Hospital and Diabetes Center, Mito, JPN; 2 Ophthalmology, International University of Health and Welfare, Atami, JPN; 3 Ophthalmology, Kitasato University School of Medicine, Sagamihara, JPN

**Keywords:** accommodative cycloplegics effect, atropine, cycloplegic eye drops, pediatric patients, refraction examination, refractive change, young children

## Abstract

Aim: This retrospective study evaluated refractive error changes following atropine sulfate eye drop use in young children.

Methods: Patients under 12 years of age who underwent their first atropine refraction between 2001 and 2021 were included in this study. The objective refractive error changes in children who underwent a second cycloplegic refraction with the same atropine concentration within 18 months of the first refraction were assessed. Refractive errors were compared between patients with myopic (myopic group) and hyperopic (hyperopic group) changes.

Results: Among the 153 eyes that received two refractive examinations, the refractive error changed by 0.21±0.61 diopters (D). The mean refractive error changes were -0.45±0.34 D and 0.49±0.46 D for the myopic (50 eyes, 33%) and hyperopic (103 eyes, 67%) groups, respectively. The mean refractive errors of the initial refractions were 5.42±2.95 D and 4.18±2.68 D for the myopic and hyperopic groups, respectively (p<0.01).

Conclusion: The refractive error changes between the first and second examinations were hyperopic, and in 67% of cases, the refractive error was possibly underestimated during the first examination.

## Introduction

Determining the refractive error using cycloplegic eye drops is essential for the diagnosis and treatment of amblyopia and strabismus in children; the most common cycloplegic eye drops are cyclopentolate hydrochloride (cyclopentolate) and atropine sulfate (atropine) [1]. The American Academy of Ophthalmology guidelines state that the use of cycloplegic eye drops is both safe and effective in pediatric patients. Moreover, they recommend the administration of cyclopentolate 0.5% in infants and cyclopentolate 1% in children aged >1 year [2]. However, atropine has been recommended as an effective cycloplegic agent for children aged five years or younger with dark irises [2,3]. Since most Japanese children have dark irises, atropine is generally preferred for cycloplegia in this population.

Atropine has a stronger cycloplegic effect than cyclopentolate [4-8]. Sani et al. reported a change in refractive error of -0.19 D when 1% cyclopentolate and 1% tropicamide eye drops were instilled five weeks after 1% atropine eye drops [6]. Fan et al. observed a change in refractive error of +0.42 D when 1% atropine eye ointment was instilled two weeks after 1% cyclopentolate and 1% tropicamide eye drops [3]. Hyperopia in accommodative esotropia is known to increase for three years after the prescription of glasses and then either increase or remain unchanged for the next seven years [8-12]. In addition, moderate hyperopia can persist until the age of seven years in children with hyperopic infantile esotropia [13]. However, a continuous decrease in hyperopia over time has been reported in accommodative esotropia [14,15].

Patients who undergo repeated cycloplegic refractions often have higher levels of hyperopia at the second examination. However, previous findings are primarily based on patients with accommodative or infantile esotropia, and studies on hyperopic patients without strabismus remain limited. Furthermore, there are no reports on the use of atropine alone. Therefore, this study compared the refractive changes in patients who underwent two consecutive atropine cycloplegic refractions.

## Materials and methods

Study approval

This study adhered to the principles outlined in the Declaration of Helsinki. Ethical approval was obtained from the Ethics Review Board of Kozawa Eye Hospital and Diabetes Center, Mito City, Ibaraki, Japan, in May 2022 (approval number: KG2022-22). Informed consent was obtained from the parents/guardians of the participants through an opt-out option made available on the Kozawa Eye Hospital and Diabetes Center website.

Study design

We retrospectively included patients aged six months to 12 years who first underwent cycloplegic refraction using atropine as the cycloplegic agent between September 2001 and December 2021 at Kozawa Eye Hospital and Diabetes Center. Changes in refractive errors were examined in patients who underwent a second cycloplegic refraction using the same atropine concentration within 18 months of the initial atropine refraction.

We excluded patients whose initial accommodative cycloplegic was not atropine, those with discrepant atropine concentrations or autorefractors that differed between the first and second atropine refractions, and those aged >13 years.

The patients received atropine eye drops twice a day for seven days. Children aged <6 years received 0.5% atropine, and children aged ≥6 years received 1.0% atropine; however, some children aged <6 years received 1.0% atropine owing to strabismus or amblyopia. The patients who underwent a second atropine refraction were administered the same concentration of atropine drops as in the first examination. When the doctor prescribed the eye drops, a prepared instruction sheet was provided that explained the reason for using the drops, how to use them, and their potential effects and adverse reactions, such as fever and flush. Specifically, the parents/guardians were asked to adhere to the following steps: pressure should be applied to the lacrimal sac for 1 min after administering each drop; only one drop should be administered at a time and additional drops should not be administered; and if the patient feels unwell after starting the eye drops or develops adverse reactions, the treatment should be discontinued and the parent/guardian should contact the hospital.

All atropine refractive error measurements were objectively obtained from autorefractors (Aichi, Japan: Tomey) and handheld refractors (Tokyo, Japan: Right), limited to the right eye. Astigmatism was determined using the spherical equivalent (SE). Patients with a refractive change difference between the two examinations during atropine use of <0 diopters (D) and ≥0 D were categorized into the myopic and hyperopic change groups, respectively. The hyperopic change group was further subdivided into the following three subgroups based on the magnitude of refractive error change: 0 to <0.50 D, 0.50 to <1.00 D, and ≥1.00 D. Hyperopia was classified as mild/moderate (<5.00 D) or high (≥5.00 D).

In this study, we analyzed objective refractive error changes between the first and second atropine refractions, the interval between the two atropine refractions in the myopic and hyperopic groups, the first SE value, age at the first eye drop administration, and a comparison of the first SE value and age at first eye drop administration among the three hyperopic change subgroups. The SE values for the first refraction were compared by the refractive error type.

Data analyses

All statistical analyses were performed using EZR (Easy R; Saitama, Japan: Saitama Medical Center, Jichi Medical University), which is a graphical user interface for R (Vienna, Austria: R Foundation for Statistical Computing), a modified version of R Commander designed to include the statistical functions frequently used in biostatistics [15]. The Shapiro-Wilk normality test confirmed that the data conformed to a non-normal distribution. Statistical processing was performed using the Wilcoxon signed-rank test and the Mann-Whitney U test for between-group comparisons, and the Kruskal-Wallis test for among-group comparisons. A p-value ≤0.05 was considered significant.

## Results

Atropine refraction findings

**Figure 1:**
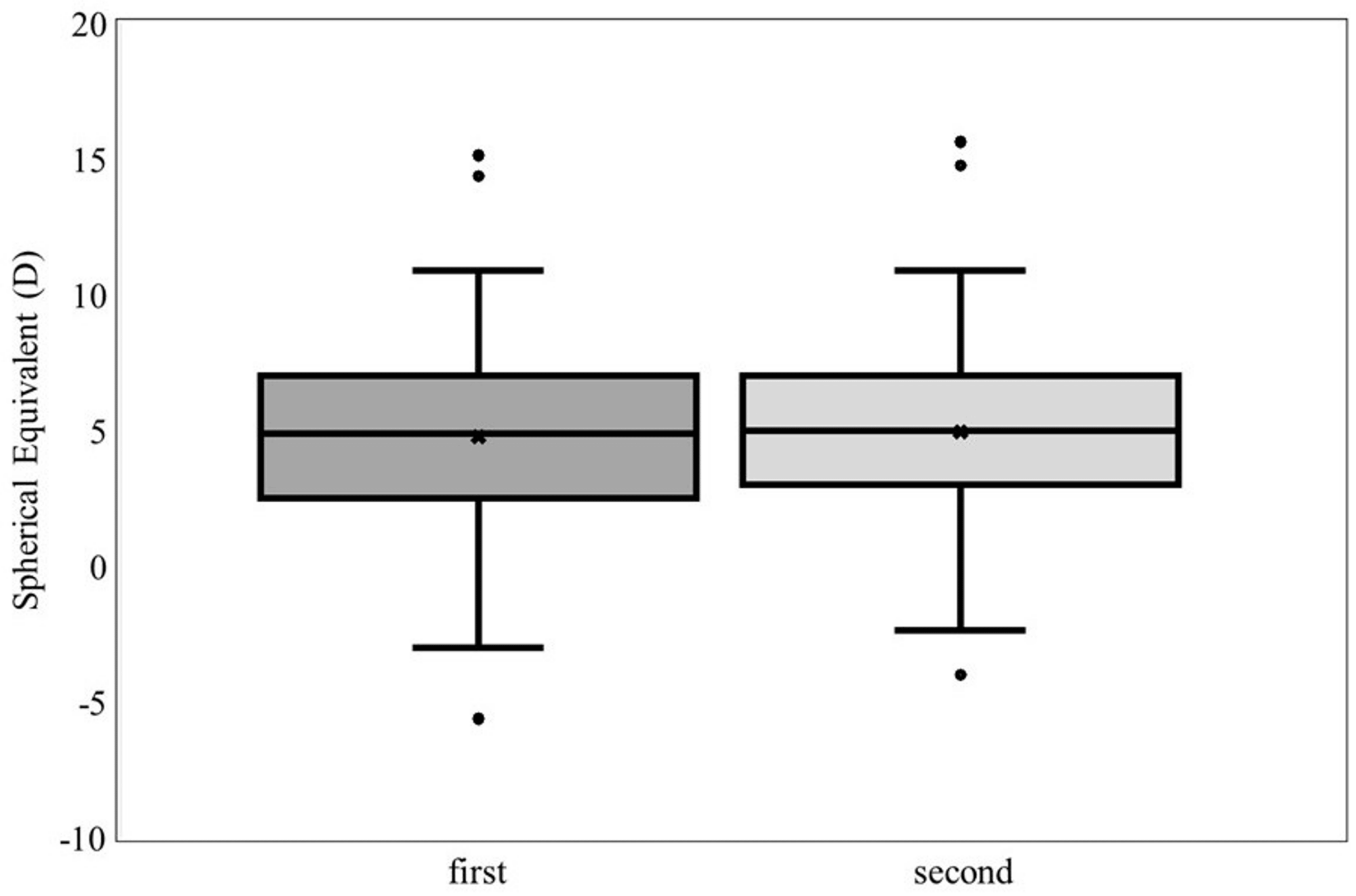
Comparison of atropine refraction between the first and second examinations. The mean SE values for the first and second atropine refractions were 4.59±2.83 D and 4.79±2.72 D, respectively. Increased hyperopia was found at the second atropine refraction, and the mean refractive error increased by 0.21±0.61 D (95% CI: 0.11-0.31 D; p<0.01). CI: confidence interval; D: diopters; SE: spherical equivalent

Two atropine refractions were conducted in 153 eyes (85 eyes of boys and 68 eyes of girls). All patients were able to receive atropine eye drops as indicated. The mean age at the first examination was 4.31±1.68 years (4.43±1.36 years for boys and 4.16±1.99 years for girls). Increased hyperopia was found on average across all patients at the second atropine refraction, and the mean refractive error increased by 0.21±0.61 D (95% CI: 0.11-0.31 D; p<0.01) (Figure 1).

Changes in refractive error during two atropine refractions

**Figure 2:**
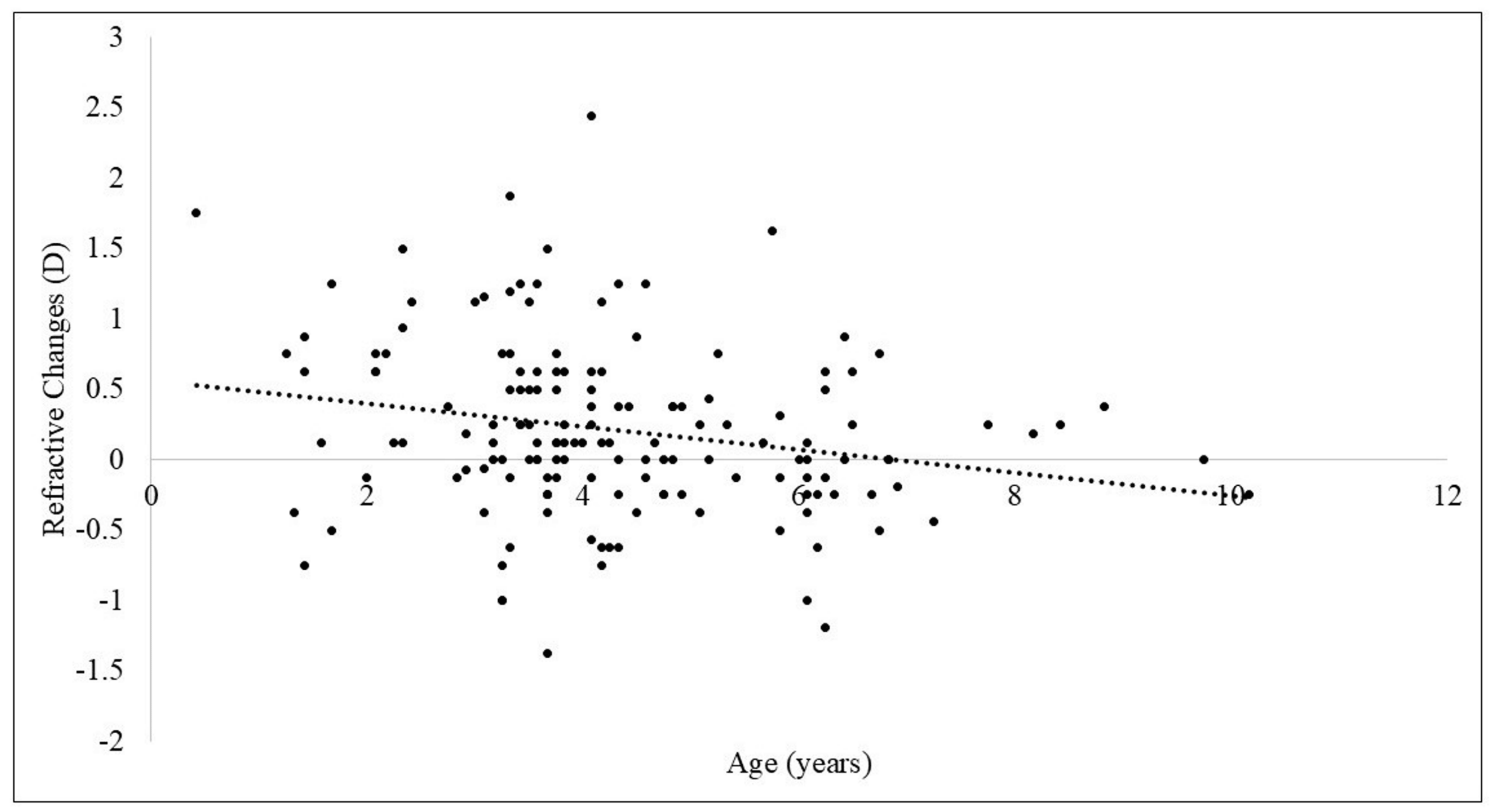
Distribution chart with refractive changes and the age at the administration of the first atropine eye drops. The slope coefficient of the regression equation is close to zero. Pearson's product-moment correlation shows almost no correlation (y = -0.0812x + 0.5599, r = -0.22, 95% CI: -0.37 to -0.07, p<0.01). CI: confidence interval; D: diopters

The mean refractive error changes were -0.45±0.34 D and +0.49±0.46 D in the myopic (50 eyes) and hyperopic (103 eyes) groups, respectively. The second atropine refraction strongly elicited a greater degree of hyperopia in 67% of patients. The SE values for the first atropine refraction examination were 5.42±2.95 D and 4.18±2.68 D in the myopic and hyperopic change groups, respectively; the SE value in the hyperopic change group was lower by 1.24 D (p<0.01) (Table 1). Figure 2 shows the distribution chart with refractive changes and the patients’ age at the administration of the first atropine eye drops. The slope coefficient of the regression equation was close to zero.

**Table 1:** Results of the two atropine refractions in the myopic and hyperopic changes. *P<0.01 was significant. Differences were evaluated using the Mann-Whitney U test. CI: confidence interval; D: diopter; SD: standard deviation; SE: spherical equivalent

Refraction value change		Myopic change (<0 D)	Hyperopic change (≥0 D)	W-value	p-Value
		n=50	n=103		
Interval between examinations	Mean months±SD (95% CI)	9.33±4.29 (8.11-10.55)	9.52±3.87 (8.76-10.28)	2478	0.71
SE at the first atropine refraction	Mean D±SD (95% CI)	5.42±2.95 (4.58-6.26)	4.18±2.68 (3.66-4.70)	3328	<0.01*
SE at the second atropine refraction	Mean D±SD (95% CI)	5.01±2.94 (4.17-5.85)	4.69±2.60 (4.18-5.20)	2881	0.24
Age in years at first eye drop	Mean years±SD (95% CI)	4.58±1.68 (4.10-5.06)	4.18±1.66 (3.86-4.50)	2943	0.15

Hyperopic change group

The hyperopic change group was divided into the following three subgroups: 0 to <0.50 D (56 eyes), 0.50 to <1.00 D (30 eyes), and ≥1.00 D (17 eyes). The mean ages at the first examination were 4.69±1.53 years, 3.72±1.53 years, and 3.33±1.17 years, respectively (p<0.01); the 0 to <0.50 D group was significantly older than the 0.50-1.00 D and >1.00 D groups (p=0.02, p<0.01, respectively) (Table 2).

**Table 2:** Hyperopic changes in the three subgroups. *P<0.01 was significant. Differences were evaluated using the Kruskal-Wallis test. CI: confidence interval; D: diopters; SD: standard deviation; SE: spherical equivalent

Refraction value		0 to <0.50 D	0.50 to <1.00 D	≥1.00 D	H-value	p-Value
		n=56	n=30	n=17		
First SE (D)	Mean D±SD	4.59±2.14	3.85±2.14	3.42±3.85	3.64	0.16
	95% CI	4.02-5.16	3.05-4.65	1.44-5.40		
Age in years at first eye drop	Mean years±SD	4.69±1.53	3.72±1.53	3.33±1.17	12.42	*<0.01
	95% CI	4.28-5.10	3.15-4.29	2.73-3.93		

Classification based on refractive errors

**Figure 3:**
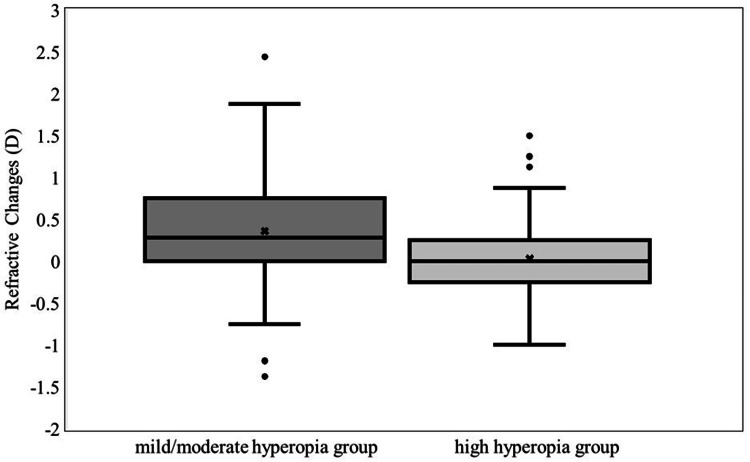
Comparison of two atropine refractions in refractive errors of hyperopia. The mean refractive error changes in the mild/moderate and high hyperopia groups were 0.36±0.64 (95% CI: 0.21-0.51) D and 0.03±0.52 (95% CI: -0.08 to 0.14) D, respectively. The refractive error change in the high hyperopia group decreased by 0.33 D (p<0.01). CI: confidence interval; D: diopters

Among the 153 eyes with hyperopia, the refractive error was mild to moderate in 71 eyes (range: 0.125-4.88 D) and high in 82 eyes (range: 5.00-14.38 D). The change in refractive error in the high hyperopia group after two atropine refractions was 0.33 D less than that in the mild to moderate hyperopia group (p<0.01) (Figure 3).

## Discussion

The mean refractive change in patients who underwent two atropine refraction examinations was 0.21±0.61 D, with hyperopia observed in 67% of cases. Notably, for some patients, it was difficult to obtain maximum hyperopia in the initial atropine refraction.

Previous studies have reported that when a second cyclopentolate refraction was performed following the initial atropine refraction, refraction values shifted from -0.19 to -0.31 D [6,7]. Fan et al. demonstrated an increase in hyperopia of 0.42 D when refraction was measured with atropine eye ointment two weeks after cyclopentolate eye drops [3]. In this study, although the mean refractive change shifted toward hyperopia, the magnitude of change was smaller than that found in their study. This discrepancy may be attributed to the use of atropine in both examinations and the maximum interval of 18 months between the first and the second atropine doses. Atropine is considered the highest cycloplegic agent among those studied [3,5-7]. The hyperopic change at the second atropine refraction was observed in 67% (103/153 eyes) of patients. These results indicate that some patients did not achieve a sufficient accommodative cycloplegic effect after the first atropine eye drop administration. Previous studies have reported that hyperopia either increased or remained unchanged in patients with accommodative esotropia following refraction examination with 1% cyclopentolate [8-13]. This increase in hyperopia is likely due to the gradual relaxation of the ciliary muscle in response to the optical correction [16]. However, the average refractive change of 0.21 D observed in this study might not be clinically significant. Furthermore, the use of emmetropization might reportedly initially worsen and subsequently improve hyperopia [17]. Notably, the current results might include natural aging-associated hyperopia. However, as presented in Figure 2, the regression equation is close to zero, suggesting that age minimally affected emmetropization in this study. Furthermore, numerous previous studies have indicated that cycloplegic eye drops should be administered to accurately measure refractive values, making the use of cycloplegics necessary [18-20].

When the participants were divided into myopic and hyperopic change groups based on refractive error changes, the first SE value was 1.24 D lower for the myopic change group than for the hyperopic change group. Moreover, the average age at the time of the first eye drop administration was 0.4 years younger for the hyperopic change group than for the myopic change group. The subgroup with a hyperopic change of >1.00 D had an initial SE of 3.42 D, and the mean age at first eye drop administration was 3.33 years.

Fan et al. demonstrated refractive changes of 0.55 D and 0.28 D after administration of 1% cyclopentolate followed by atropine in children aged ≤5 years and >5 years, respectively [3]. Similarly, Farhood showed differences in refractive change between cyclopentolate and atropine of 0.40 D and 0.20 D in the ≤5 and >5 age groups, respectively [7]. In addition, Bonafede et al. found that among children aged 3-7 years, hyperopia increased by 0.12 D/year in those with baseline hyperopia <4 D, whereas it remained stable in those with baseline hyperopia ≥4 D [9]. In the present study, the group with hyperopic changes was younger, as was the group with >1 D of refractive error that shifted toward hyperopia. These results suggest that in younger children, initial refraction using atropine may not be possible to maximally extract hyperopia.

When the initial SE values were compared between the groups with mild/moderate hyperopia of <5.00 D and high hyperopia of ≥5.00 D, the refractive error changes were 0.36 D and 0.03 D, respectively, and hyperopia was induced more strongly by the second instillation in eyes with mild/moderate hyperopia. Our study results suggest that patients with a first SE measurement of ≤5.00 D are unlikely to obtain a sufficient accommodative cycloplegic effect. The first SE measurement in the group, in which hyperopia was more strongly induced by the second atropine dose, was 3.42 D; their mean age at first eye drop instillation was 3.33 years. If the first SE measurement is approximately 3.00 D or when the patient is approximately three years old, the second eye drop may induce more hyperopia.

The limitations of this study include its retrospective design, the omission of strabismus as a variable, the natural progression of hyperopia associated with aging, and the lack of standardization in examination equipment, given that the study period spanned 20 years. However, each individual patient was assessed using the same measurement instrument. We believe that inter-instrument variability was minimal because Karabulut et al. reported a strong correlation between the equivalent spherical frequencies obtained from both instruments [21].

## Conclusions

The results of this study suggest that the initial atropine cycloplegic refraction in young children below the age of 12 years might underestimate the degree of hyperopia, particularly in younger children and those with an initial SE of approximately +3.00 D. Therefore, patients in this high-risk group should be carefully followed up or undergo a second cycloplegic examination and the initial results should not be solely relied upon. The results of our study have important clinical implications for the use of atropine cycloplegia in refractive error evaluation in young children.
